# Does hyperbaric oxygen cause narcosis or hyperexcitability? A quantitative EEG analysis

**DOI:** 10.14814/phy2.15386

**Published:** 2022-07-20

**Authors:** Xavier C. E. Vrijdag, Hanna van Waart, Chris Sames, Simon J. Mitchell, Jamie W. Sleigh

**Affiliations:** ^1^ Department of Anaesthesiology University of Auckland Auckland New Zealand; ^2^ Slark Hyperbaric Unit Waitemata District Health Board Auckland New Zealand; ^3^ Department of Anaesthesia Auckland City Hospital Auckland New Zealand; ^4^ Department of Anaesthesia Waikato Hospital Hamilton New Zealand

**Keywords:** complexity, diving, EEG, functional connectivity, oxygen narcosis

## Abstract

Divers breathe higher partial pressures of oxygen at depth than at the surface. The literature and diving community are divided on whether or not oxygen is narcotic. Conversely, hyperbaric oxygen may induce dose‐dependent cerebral hyperexcitability. This study evaluated whether hyperbaric oxygen causes similar narcotic effects to nitrogen, and investigated oxygen's hyperexcitability effect. Twelve human participants breathed “normobaric” air and 100% oxygen, and “hyperbaric” 100% oxygen at 142 and 284 kPa, while psychometric performance, electroencephalography (EEG), and task load perception were measured. EEG was analyzed with functional connectivity and temporal complexity algorithms. The spatial functional connectivity, estimated using mutual information, was summarized with the global efficiency network measure. Temporal complexity was calculated with a “default‐mode‐network (DMN) complexity” algorithm. Hyperbaric oxygen‐breathing caused no change in EEG global efficiency or in the psychometric test. However, oxygen caused a significant reduction of DMN complexity and a reduction in task load perception. Hyperbaric oxygen did not cause the same changes in EEG global efficiency seen with hyperbaric air, which likely related to a narcotic effect of nitrogen. Hyperbaric oxygen seemed to disturb the time evolution of EEG patterns that could be taken as evidence of early oxygen‐induced cortical hyperexcitability. These findings suggest that hyperbaric oxygen is not narcotic and will help inform divers' decisions on suitable gas mixtures.

## Graphical abstract

Visual summary of the study. 12 participants were exposed to normobaric air and oxygen, before pressurised to 1.4 and 2.8 ATA (in random order). EEG Connectivity did not show a change, while complexity was reduced during oxygen breathing.

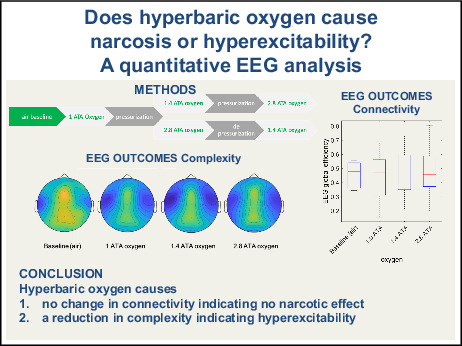

## 1 INTRODUCTION

To sustain life, every breathing gas mixture for divers must include oxygen and divers invariably breathe oxygen at partial pressures higher than in air at one atmosphere. Hyperbaric oxygen has been postulated to cause both narcosis (Lang, 2006; Linnarsson et al., 1990), a state of cognitive impairment (Frankenhaeuser et al., 1963; Freiberger et al., 2016; Hesser et al., 1978), and hyperexcitability (Bitterman, 2004; Ciarlone et al., 2019; Rostain & Lavoute, 2016), a state of increased neuronal activity with enhanced function (Germonpré et al., 2017; Scholey et al., 1999). Some authors even suggest hyperbaric oxygen is capable of producing both states, although with a time delay of 30 min for hyperexcitability to occur as part of central nervous system (CNS) oxygen toxicity (Kot et al., 2015). Others have found no cognitive change (Bennett et al., 1969; Frankenhaeuser et al., 1960; Gill et al., 2014).

A study using electroencephalography (EEG), found no effect of 100% oxygen at 284 kPa for 30 Minutes (Visser et al., 1996), while a 10‐min period of air‐breathing at the same pressure did increase alpha peak frequency. This indicated that oxygen is not narcotic in a similar way to nitrogen, but these measurements were performed in 1996 when EEG technology was less well developed. A more recent and sophisticated study, also exposing divers to 284 kPa for 20 min, showed an increase in posterior alpha power combined with a decrease in delta power starting after 5 min of oxygen breathing and reaching its minimum at 20 min (Pastena, Formaggio, Faralli, et al., 2015). This was reported as a fronto‐parietal disconnection based on source localization analysis (Storti et al., 2015). These results were attributed to oxygen hyperexcitability. Normobaric hyperoxia has been shown to decrease alpha and beta power (Kizuk et al., 2019; Sheng et al., 2017), although another study found a temporal increase in alpha power (Damato et al., 2020).

The effects of gas narcosis increase with a diver's depth, as the ambient pressure and the partial pressure of the individual gases in the breathing mix increase. Gas narcosis has traditionally been linked to nitrogen when air is respired (Behnke et al., 1935). The narcosis is causing behavioral changes and impaired cognitive abilities. Substituting helium wholly or partly for nitrogen ameliorates the narcosis effects (Mitchell & Doolette, 2013). Similarly, it has been argued that replacing some nitrogen with oxygen also reduces a gas mixture's narcotic potency (Lowry, 2002; Navy, 2018). In contrast, others believe that oxygen has a similar narcotic potency to nitrogen, and hence oxygen‐enriched mixtures have the same narcotic potency as air (Lang, 2006; Linnarsson et al., 1990). Thus, the narcotic potency of oxygen is a debated subject without a clear answer (Bennett & Rostain, 2003; Freiberger et al., 2016; Gill et al., 2014).

The relative narcotic potencies of nitrogen and oxygen are complicated to predict. The Meyer–Overton correlation indicates a proportional relationship between the potency of anesthetic gases and their solubility in liquid oils (Meyer, 1899; Overton, 1901) although there are exceptions to this; some highly hydrophobic gases are not anesthetic (Weir, 2006). Nevertheless, the abovementioned argument that oxygen is equally or more narcotic than nitrogen (Lang, 2006; Linnarsson et al., 1990) is based on the higher solubility of oxygen in both oil and water (Battino et al., 1968; Ding et al., 2014; Langø et al., 1996). However, there are other relevant considerations. Nitrogen is a non‐metabolic gas, meaning that the inhaled PN_2_, equilibrates with the PN_2_ in cerebral tissue after a short wash‐in period (Mitchell & Doolette, 2009). In contrast, oxygen is a metabolic gas consumed in the tissues, thus lowering the local PO_2_ below what would be predicted for an inert gas at an equivalent inspired pressure. Nevertheless, when arterial PO_2_ is increased, the PO_2_ in cerebral tissue does increase (Weenink et al., 2013). It follows that the higher cerebral PO_2_ seen in diving could (in theory) have a narcotic effect that should not be neglected (Bennett & Rostain, 2003).

Measuring comparative cognitive effects of hyperbaric oxygen and nitrogen is a difficult undertaking. Ideally, the comparison should take place at equivalent inspired pressures of the two gases. However, nitrogen narcosis typically occurs at inspired partial pressures greater than 300 kPa, at which oxygen‐breathing would carry the risk of cerebral oxygen toxicity (Bitterman, 2004). It follows that the measurement method needs to be sensitive enough to detect the subtle effects of gas narcosis in the more limited and safe inspired pressure range of oxygen.

A pressure of 284 kPa is considered the maximum safe inspired oxygen pressure in routine hyperbaric oxygen treatments with resting patients, conducted in dry recompression chambers (Smerz, 2004). To quantify cortical functional connectivity, our group previously developed a mutual information‐based global efficiency EEG metric which detected dose‐dependent change during air‐breathing at 284 and 608 kPa, not seen during breathing of oxygen‐helium mixes containing the same fraction of oxygen as air. These changes were therefore likely caused by the known subtle narcotic effect of nitrogen breathed at these pressures (Vrijdag et al., 2022). All subjects in that study showed some decrement in psychometric performance when breathing hyperbaric nitrogen (air at 608 kPa), but the median decrement was not statistically significant due to an unfortunate lack of sensitivity of the chosen test, a small sample size, and (possibly) an ability among motivated subjects to overcome subtle narcosis through close focus on the task. Similarly, in a dive at 24 m (344 kPa) psychometric testing only showed a very marginal difference between air and oxygen enriched‐air (nitrox 28%) in 108 divers (Brebeck et al., 2017). Nevertheless, if oxygen were to have similar narcotic effects to nitrogen, it would be expected that global efficiency would change similarly during oxygen exposure at this pressure.

As a complement to estimating spatial connectivity, the regularity of the time‐evolution of brain patterns can be quantified using a default mode network (DMN) complexity analysis that has previously proved sensitive to the narcotic effects of increasing doses of nitrous oxide (Vrijdag et al., 2021), presumably caused by modulation of neuronal NMDA receptors (Hirota, 2006). Since nitrous oxide typically causes EEG excitation co‐existing with a complex mixture of behavioral depression and euphoria manifest as “narcosis” (Jevtović‐Todorović et al., 1998), the DMN complexity analysis could potentially measure a comparable hyperexcitability tendency of oxygen.

Using these quantitative EEG algorithms, this study aimed to determine if oxygen causes similar narcotic effects on the EEG to nitrogen, and to investigate oxygen's potential hyperexcitability effect.

## 2 MATERIALS AND METHODS

### 2.1 Trial design and participants

This randomized cross‐over trial took place at the Slark hyperbaric unit, Waitemata District Health Board, from June to August 2019. The study protocol was approved by the Health and Disability Ethics Committee, Auckland, New Zealand (reference 16/NTA/93), and was registered with the Australian New Zealand Clinical Trial Registry (ANZCTR: U1111‐1181‐9722, http://www.anzctr.org.au/, RRID:SCR_002967). The sample size was based on similar studies previously published (Biersner et al., 1977; Fowler et al., 1983; Hamilton et al., 1992; Vrijdag et al., 2021).

Participants were healthy certified technical divers aged between 18 and 60 years. Candidate participants currently using recreational drugs, tobacco, psychoactive medication, excessive alcohol, or over five caffeine‐containing beverages a day were excluded. Prior to each hyperbaric exposure, participants had at least 6 h of sleep, abstained from any caffeinated drink on the day and refrained from diving and alcohol for 24 h prior. All participants provided written informed consent.

### 2.2 Experimental procedures

**FIGURE 1:**
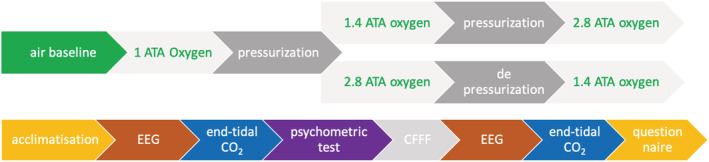
Oxygen measurements. Top row describes the order of steps during the oxygen measurement session. Measurements were conducted at air baseline, 100% oxygen measurement at 101, 142, and 284 kPa, with the last two in randomized order. The bottom row represents the recording order at each exposure. During each measurement, EEG, end‐tidal CO_2,_ psychometric test, critical flicker fusion frequency (CFFF), and again EEG and end‐tidal CO_2_ were recorded. Each measurement was finished with the NASA task load index and Karolinska sleepiness score questionnaires. Air baseline measurement did not have an acclimatization step.

All measurements were conducted inside a cylindrical 5‐person hyperbaric chamber (W.E. Smith Engineering PTY LTD). In the experimental session, participants underwent four EEG measurements. First, a baseline measurement while breathing air at surface pressure. Second, a measurement while breathing 100% oxygen at surface pressure. The third and fourth measurements took place in block‐randomized non‐blinded order at 142 and 284 kPa (Figure 1). 142 kPa was chosen as it approximates the upper limit of PO_2_ exposures accepted in diving, while 284 kPa is the maximum exposure to oxygen inside a hyperbaric chamber with an acceptable risk of oxygen toxicity. Oxygen was administered via a mouthpiece mounted on a demand regulator with a system to vent exhaled gas outside the chamber. Participants used a clip to seal the nose. Air was breathed from the chamber environment. All measurements were made after a 5‐min acclimatization period.

The experimental measurements were made in the following order (with approximate time): EEG recording (1‐min eyes open and 1‐min eyes closed), end‐tidal carbon dioxide measurement (~30 s), psychometric testing with a math‐processing task (~2 min), critical flicker fusion frequency (CFFF, this and end‐tidal carbon dioxide results published elsewhere; Vrijdag et al., 2020) (~2 min), repeat of EEG recording (2 min), and end‐tidal carbon dioxide measurement (~30 s) and finally the NASA task load index and Karolinska sleepiness scale questionnaires (~1 min) (Figure 1). The exact times varied slightly between exposures and participants. The total exposure time at each of the pressures was approximately 15 min.

### 2.3 Outcomes

EEG was recorded using a portable active electrode 32‐channel system (ActiveTwo, BioSemi) divided over the scalp based on the international 10–10 system (Klem et al., 1999) with two additional electrooculogram (EOG) electrodes placed under the eyes. The offset (impedance equivalent for active systems) was below 25 mV with gelling (SignaGel, Parker Laboratories). Signals were sampled at 1024 Hz. For analysis, the resting‐state eyes‐open segment of the second EEG sample (after the psychometric tests, Figure 1) was used, as prolonged gas exposure had occurred, and fewer artifacts were present in these recordings. After each EEG recording, end‐tidal carbon dioxide was measured by breathing through a mainstream capnograph (EMMA, Masimo).

The math processing test (psychology experimental building language [PEBL]) (Mueller & Piper, 2014) was administered on a diving computer (Icon, Mares s.p.A.) (Germonpré et al., 2017) and consisted of 20 questions with a maximum response time of 5 s. It stored reaction time and error rate (accuracy) of each question. The test was practiced until the results were stable in a training session. A combined ‘psychometric‐impairment’ metric (scaled 0–1) was calculated, averaging the cleaned, normalized (divided by the maximum) mean reaction time, and error rate.

The NASA task load index measured subjective experience of perceived work on six scales of mental demand, physical demand, temporal demand, performance, effort, and frustration level (Hart & Staveland, 1988). The Karolinska sleepiness scale asked for subjective wakefulness level (Åkerstedt & Gillberg, 1990). Both were administered on paper.

### 2.4 Quantitative EEG analysis

The dataset was cleaned and further analyzed using spectral power analysis, the mutual information‐based global efficiency metric, and the DMN complexity metric, whose details can be found in references (Vrijdag et al., 2021, 2022). In brief, the functional connectivity between each electrode is calculated using mutual information of the amplitude envelope for the alpha frequency band, to produce a connectivity matrix; from which the global efficiency was calculated using standard graph theory formulae. The DMN complexity is derived by quantifying the variability in the repetition of EEG states. A high entropy (complexity) indicates the presence of a mixture of slowly‐ and quickly‐evolving brain states, whereas a decrease in complexity is a sign that the diversity of evolution of brain states has decreased. Usually, there is a loss of slowly evolving states.

#### 2.4.1 EEG pre‐processing

The EEG epochs were cleaned during pre‐processing to remove eye movement, muscle, and noise artifacts with the Fieldtrip toolbox (version c6d58e9 RRID:SCR_004849) (Oostenveld et al., 2011). The data for each condition and exposure were cut out of the continuous recording, re‐referenced to the average, de‐meaned, de‐trended, and resampled to 256 Hz. Line (including higher harmonic) and low‐frequency noise (<1 Hz) were filtered out. Next, independent component analysis was used to filter noise components like eye blinks, high‐frequency noise, non‐physiological noise, and bad channels. An algorithm was used to advise on the manual selection of components. The data were cut in 2‐s epochs and manually inspected for remaining artifacts, with an algorithm indicating bad segments for remaining eye blinks (correlation with the EOG channels) and muscle artifacts (based on high‐frequency content of 105–120 Hz). Whole two‐second epochs were discarded if they were marked as containing artifacts. The remaining epochs for that condition and exposure were stored for further analysis.

#### 2.4.2 Frequency power analysis

Frequency power was estimated using a multitaper Fourier transformation implemented in the Fieldtrip toolbox using a Hanning window from 1 to 100 Hz with 1 Hz increments. The average power per frequency band was calculated for: Delta (1–4 Hz), theta (4–8 Hz), alpha (8–14 Hz), and beta (14–30 Hz) for each exposure.

#### 2.4.3 Mutual information‐based global efficiency

The mutual information‐based global efficiency metric starts with re‐referencing using a Hjorth derivation, narrowband filtering, and Hilbert transform; functional connectivity between the 32 channels was calculated for the alpha frequency band using the mutual information method as implemented in the information breakdown toolbox (ibtb) incorporated in Fieldtrip (Magri et al., 2009). The connectivity matrix was binarized based on an individual threshold of 80% of the range of the air baseline matrix. Global efficiency was calculated as summary network statistics, based on the node distance, also implemented in the fieldtrip toolbox (Rubinov & Sporns, 2010).

#### 2.4.4 DMN complexity metric

The DMN complexity metric (Vrijdag et al., 2021) quantifies the spatial distribution of temporal EEG complexity and is comprised of: (1) Absolute cross‐correlation calculated between consecutive 0.25 s time samples; (2) binarizing these cross‐correlation matrices using the median of all channels as threshold; (3) using quantitative recurrence analysis, the complexity in temporal changes were calculated by the Shannon entropy of the probability distribution of the diagonal line lengths. Steps 1–3 were executed for each channel separately, followed by; (4) overall spatial extent and intensity of brain complexity were quantified by calculating median temporal complexity of channels whose complexities were above one at baseline. This region approximately overlaid the medial components of the brain's default mode network.

### 2.5 Statistical analysis

All data were analyzed with Matlab version 2018a (Mathworks, RRID:SCR_001622) except the psychometric test results, the NASA task load index and the Karolinska sleepiness scale, which were analyzed with SPSS version 27 (IBM, RRID:SCR_002865). All outcome measures were tested for normality with the Kolmogorov–Smirnov (K–S) test, and subsequently characterized by their mean and standard deviation (SD). Comparisons of psychometric test results, normally distributed NASA task load index scales and both EEG metrics between the exposures were analyzed with a two‐tailed paired *t*‐test and reported as mean difference and 95% confidence intervals (CI). Comparisons of the non‐normally distributed NASA task load index and the Karolinska sleepiness scale between the exposures were analyzed with the nonparametric Wilcoxon signed‐rank test and reported as median and range. Descriptive statistics were generated to characterize the study participants. Differences were regarded as significant at *p* < 0.05, with the Bonferroni correction applied for multiple comparisons.

#### 2.5.1 Cluster‐based permutation testing

The differences between exposures in: (i) Mutual information connectivity between all channels, and (ii) the frequency power analysis, were evaluated with cluster‐based permutation testing. This method is described in more detail in Maris & Oostenveld (2007). In short, the first step clustered adjacent spatiospectral points when dependent *t*‐values exceeded the cluster threshold *p*‐value of 0.05 (two‐tailed) by summation of the *t*‐values. In the second step, a surrogate null distribution was created by randomly shuffling the condition labels 1000 times. The observed cluster‐level statistic was compared against the null distribution, and clusters with a *p*‐value below 0.05 (two‐tailed) were considered significant. Effect size (Cohen's *d*) was calculated as an average over all significant cluster nodes.

## 3 RESULTS

The 12 divers (9 male), aged between 24 and 55 years (38 ± 9), with an body mass index of 26.7 kg/m^2^ ± 4.4, reported making on average 1163 dives (range 160–3500) over 17 years (range 3–31). There was a significant decrease in end‐tidal carbon dioxide level from baseline air‐breathing to oxygen‐breathing at 142 kPa (5.3 ± 1.1 kPa at baseline to 4.6 ± 0.9 kPa, MD 0.65 [95%CI 0.25–1.05], *p* = 0.005). End‐tidal carbon dioxide levels in the other oxygen exposures were not significantly different from baseline air‐breathing.

### 3.1 Psychometric test result

**FIGURE 2:**
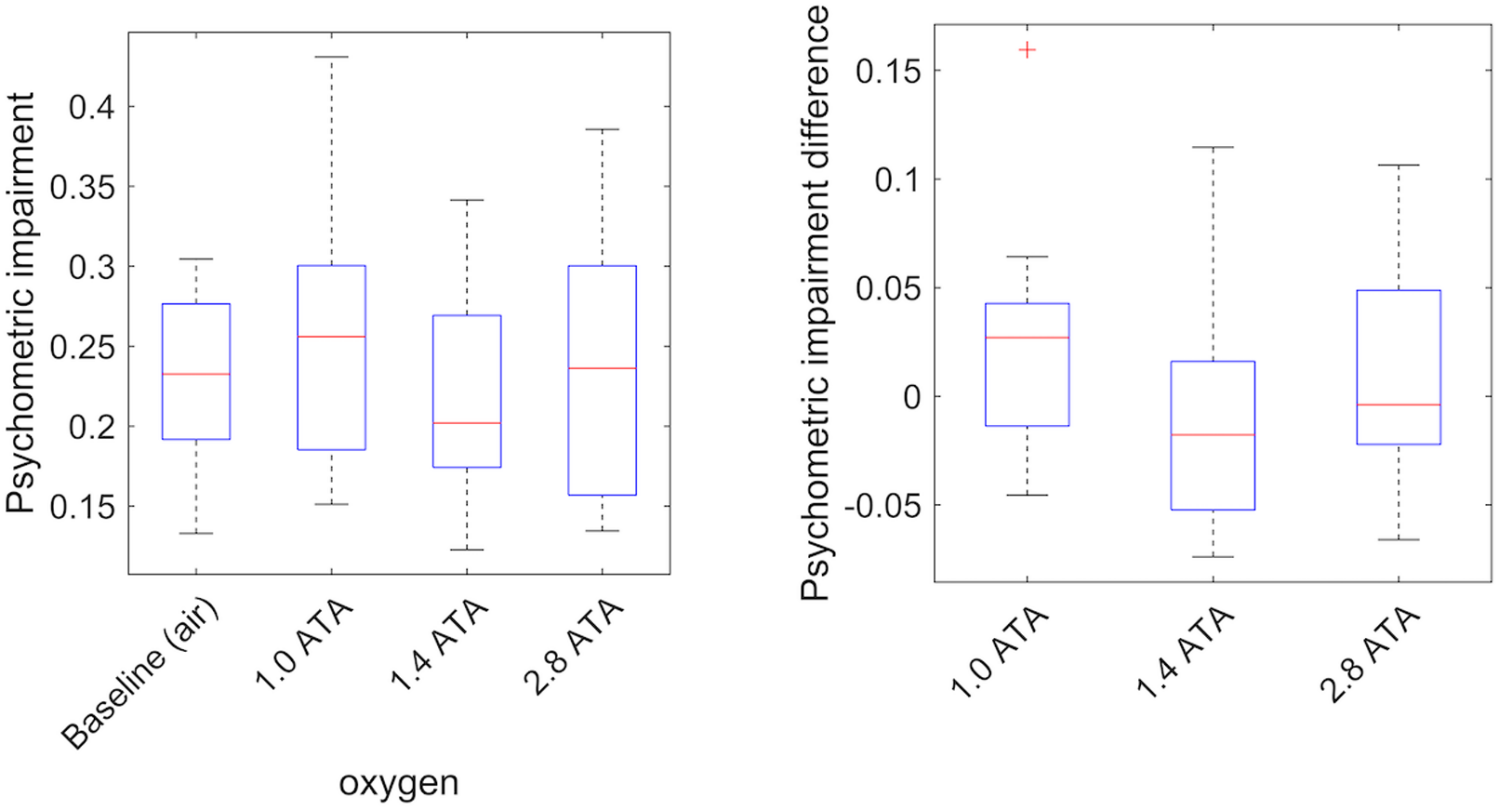
Box plot of the psychometric test results (reaction time and accuracy) combined metric (left) and the differences between exposures and air baseline (right). The box plot shows the median (red line), interquartile range (blue box) and the whiskers showing the full range of the data. Outliers (red +) are values larger than 1.5 times the interquartile range.

The psychometric test showed no significant change for any of the oxygen exposures, compared to breathing air at baseline (Figure 2).

### 3.2 Frequency power analysis

**FIGURE 3:**
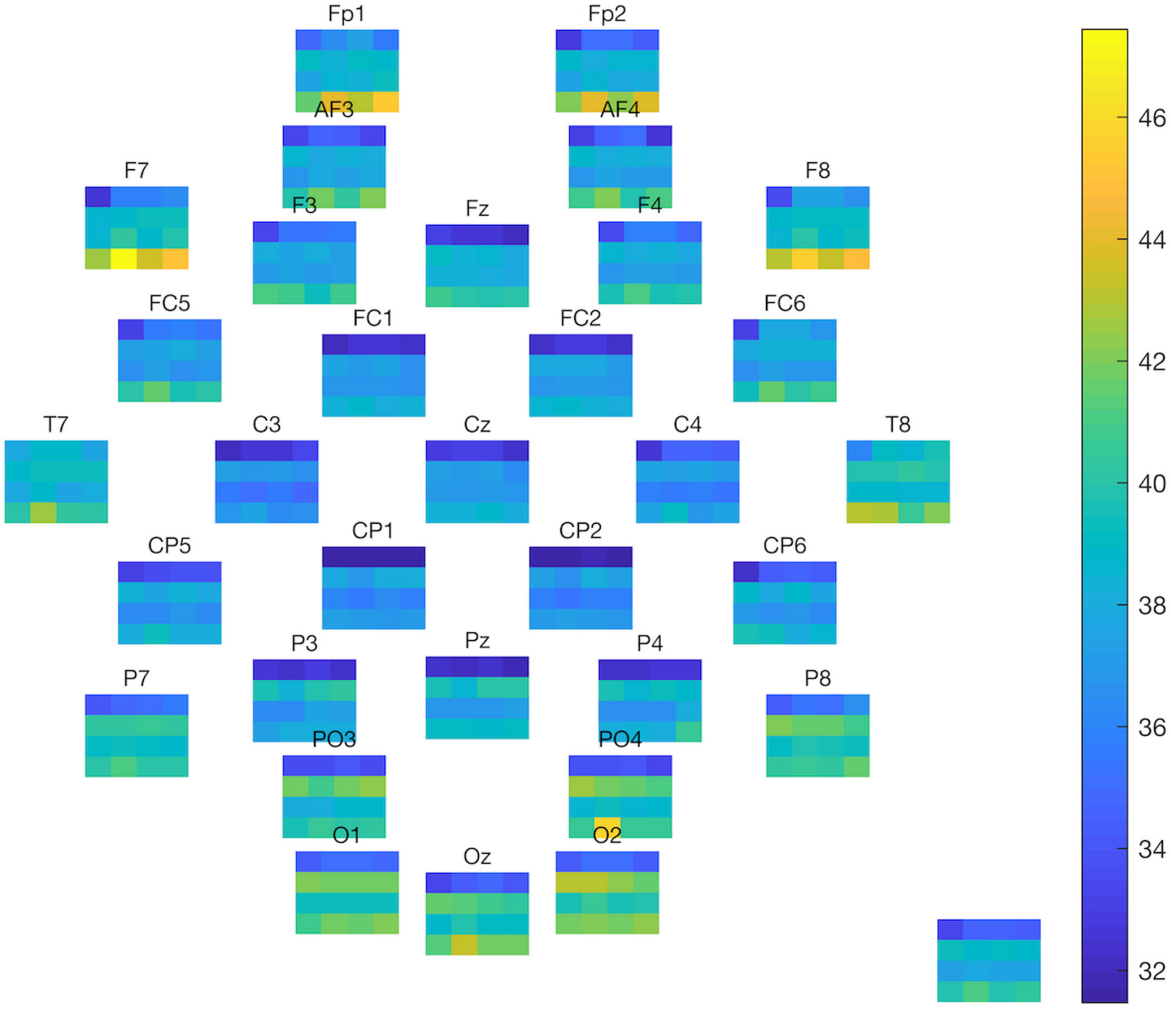
Average power in dB for each electrode (placed according to their head map location, title is the electrode name) and the mean of all electrodes (bottom right) of all participants. Each subfigure has the four exposures on the *x*‐axis (air baseline, oxygen at 101, 142, and 284 kPa) and four frequency bands on the *y*‐axis (bottom to top: Delta [1–4 Hz], theta [4–8 Hz], alpha [8–14 Hz], and beta [14–30 Hz]).

Of the 30 2‐s EEG samples on average, half a sample was removed due to muscle and eye movement artifacts. Based on permutation testing there were no significantly different clusters between baseline and any of the oxygen exposures for any of the four frequency bands (Figure 3).

### 3.3 Functional connectivity—mutual information

**FIGURE 4:**
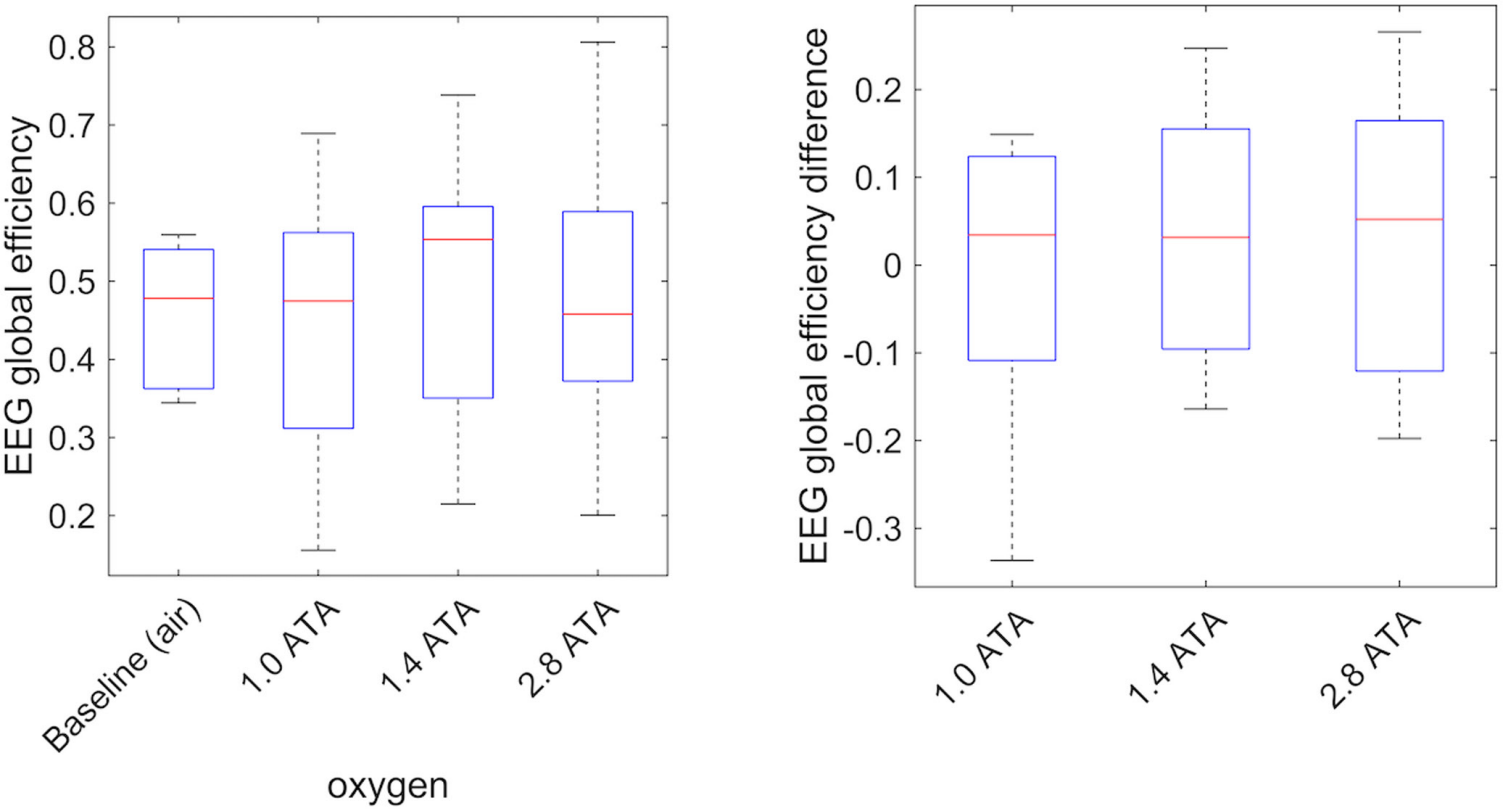
Box plot of mutual information based global efficiency metric in the alpha band (left) and the differences between exposures and air baseline (right). The box plot shows the median (red line), interquartile range (blue box), and the whiskers showing the full range of the data.

There was no significant difference in functional connectivity between baseline (air) and any of the three oxygen exposures in the global efficiency metric (Figure 4).

### 3.4 DMN complexity metric

**FIGURE 5:**
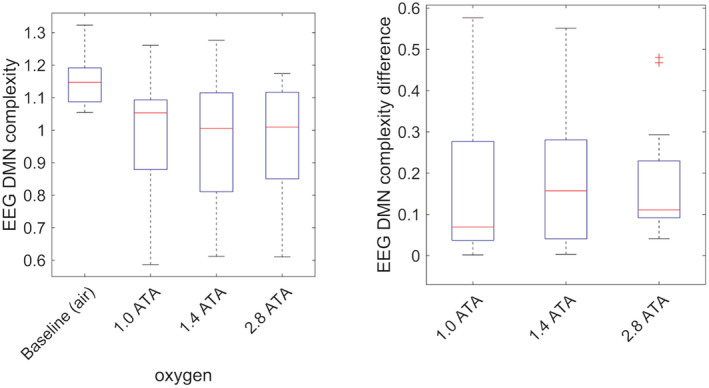
Box plot of EEG DMN complexity metric (in bits) (left) and the differences between exposures and air baseline (right). The box plot shows the median (red line), interquartile range (blue box), and the whiskers showing the full range of the data. Outliers (red +) are values larger than 1.5 times the interquartile range.

**FIGURE 6:**
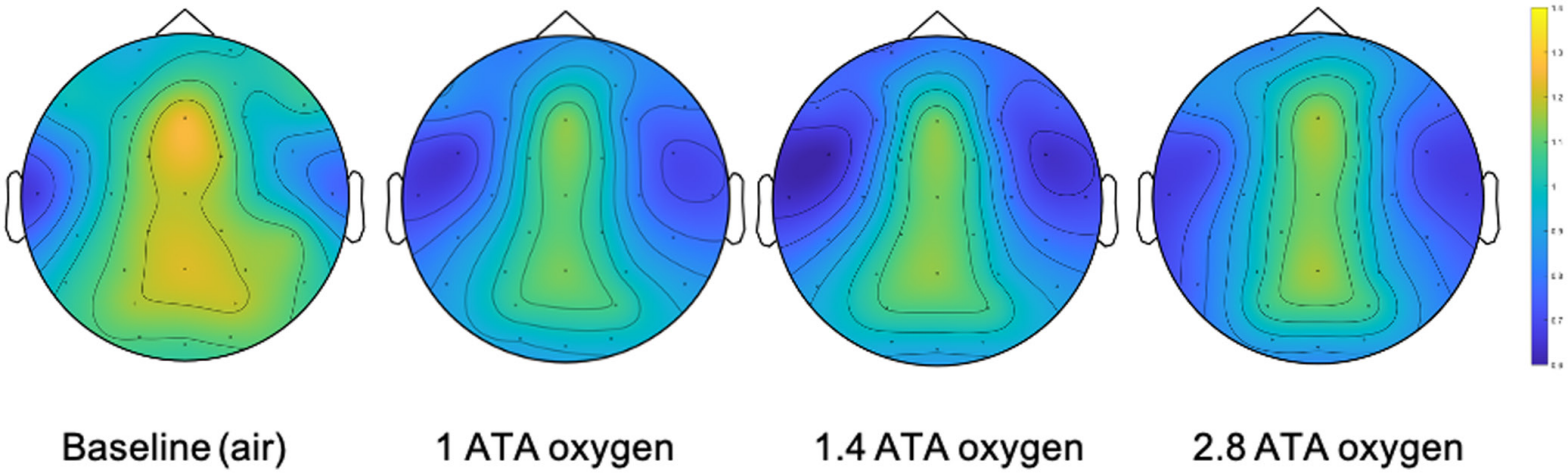
Average surface head plots with contour lines of the Shannon entropy (in bits) for all electrodes (blue low, yellow high) for each of the exposures of all participants.

The DMN complexity metric showed a non‐dose‐dependent significant decrease between baseline air breathing and the 101, 142, and 284 kPa oxygen exposures with a mean difference of 0.16 (95% CI 0.05–0.27, *p* = 0.010), 0.18 (95% CI 0.08–0.28, *p* = 0.003) and 0.18 (95% CI 0.08–0.28, *p* = 0.002), respectively (Figure 5). Evaluation of the temporal complexity head plots showed a similar pattern with a reduction in central Shannon entropy, which is consistent between the three oxygen exposures (Figure 6).

### 3.5 Subjective experience

Participants experienced a significant reduction in physical demand and effort, as scored on the NASA task load index questionnaire, for all three oxygen exposures compared to baseline air‐breathing. Additionally, there was a significant decrease in mental demand and frustration at 142 kPa compared to baseline air‐breathing (Tables 1, 2, 3).

**TABLE 1:** Mean (SD) of normally distributed NASA task load index questionnaire responses

	Baseline	1 ATA	1.4 ATA	2.8 ATA
Mental demand	27.1 (21.4)	29.2 (22.8)	33.3 (23.4)	33.8 (22.3)
Physical demand	8.8 (10.5)	15.8 (11.2)	16.3 (12.3)	21.3 (11.9)
Performance	29.2 (12.6)	34.6 (20.9)	33.8 (20.4)	32.5 (17.1)
Effort	23.3 (21.0)	30.8 (23.5)	35.4 (22.4)	38.8 (20.2)

**TABLE 2:** Mean difference (95% confidence interval) of normally distributed NASA task load index questionnaire responses while breathing oxygen at 1, 1.4, and 2.8 ATA compared to baseline

	1 ATA	1.4 ATA	2.8 ATA
Mental demand	−2.1 (−6.9 to 2.7)	−6.3 (−12.5 to 0.0)^a^	−6.7 (−14.4 to 1.0)
Physical demand	−7.1 (−10.8 to −3.4)^a^	−7.5 (−10.4 to −4.6)^a^	−12.5 (−17.1 to −7.9)^a^
Performance	−5.4 (−16.9 to 6.0)	−4.6 (−16.8 to 7.6)	−3.3 (−15.9 to 9.3)
Effort	−7.5 (−14.9 to −0.1)^a^	−12.1 (−22.1 to −2.1)^a^	−15.4 (−24.0 to −6.8)^a^

^a^ Significant difference (*p* < 0.05).

**TABLE 3:** Median (range) of non‐normally distributed NASA task load index questionnaire and the Karolinska sleepiness scale responses

	Baseline	1 ATA	1.4 ATA	2.8 ATA
Sleepiness	3.0 (2.0–6.0)	3.0 (2.0–6.0)	4.0 (2.0–6.0)	3.5 (2.0–6.0)
Temporal demand	15.0 (5.0–80.0)	22.5 (5.0–75.0)	17.5 (10.0–75.0)	25.0 (5.0–75.0)
Frustration	10.0 (5.0–70.0)	12.5 (5.0–65.0)	25.0 (5.0–80.0)^a^	22.5 (5.0–90.0)

^a^ Significant difference compared to baseline (*p* < 0.05).

## 4 DISCUSSION

This study aimed to determine if oxygen has similar narcotic properties to nitrogen, and also to investigate any potential hyperexcitability effects.

### 4.1 Oxygen narcosis

The mutual information‐based global efficiency metric of EEG spatial functional connectivity did not increase while breathing hyperbaric oxygen, in contrast to the 18% increase that we previously found in subjects breathing air at 284 kPa (Vrijdag et al., 2022). This suggests that oxygen is not producing the same EEG patterns as seen during hyperbaric air‐breathing and likely attributable to nitrogen narcosis.

Previous EEG studies of participants breathing oxygen at 284 kPa showed an excitatory effect (increased alpha power) in the posterior region (Pastena, Formaggio, Storti, et al., 2015), and a frontal–parietal disconnection compared to breathing air at surface pressure (Storti et al., 2015). In these studies, participants had their eyes closed, while in the present study, the EEG recording during eyes open was analyzed. Another study found no change in the frequency power analysis while breathing oxygen after breathing air at 284 kPa (Visser et al., 1996). This is consistent with our results, which do not show a frequency power change.

Also, our psychometric test did not show a reduction, indicating an absence of oxygen narcosis. This is similar to the results found by Lafère et al. (2019) and Hemelryck et al. (2013) even found an increase in cognitive performance during normobaric oxygen breathing in the same test as used in this study.

### 4.2 Oxygen hyperexcitability

Our study found a non‐dose‐dependent decrease in DMN temporal complexity, which was similar in magnitude to the reduction caused by 30% end‐tidal nitrous oxide (Vrijdag et al., 2021). Nitrous oxide is known to increase the excitability of the brain (Hirota, 2006). Thus, the DMN complexity reduction in the present study could be due to oxygen‐induced hyperexcitability. However, there was a dissociation between EEG complexity and psychometric impairment. Oxygen did not cause the equivalent change in cognitive impairment found in the nitrous oxide study.

On the other hand, participants did experience a subjective decrease in various scales of the task load index. This indicates that they found it easier to perform the tasks while breathing oxygen. Specifically, the effort scale showed large reductions associated with oxygen‐breathing, and could be related to mild euphoria related to hyperoxia (Bean, 1945; Sperlich et al., 2017). Cognitive enhancement by hyperoxia has been found previously in psychometric performance tests of memory encoding and stimulus reaction (Damato et al., 2020; Scholey et al., 1999, 2020). These alterations in perceived respiratory interoception could, themselves, cause changes in DMN function in a top‐down phenomenon—as has been observed in meditation and free diver breath holding (Annen et al., 2021).

A recent in vitro study demonstrated that 507 kPa of oxygen caused a significant increase in response amplitude of a specific NMDA receptor subunit (GluN2A), while lower pressures showed a linear trend toward the same effect (Bliznyuk et al., 2020). These subunits are highly expressed in the cortex and hippocampus (Sanz‐Clemente et al., 2012), and hippocampal expression is associated with learning and memory consolidation (Cercato et al., 2017). Nitrous oxide mainly inhibits NMDA‐induced amplitudes (Jevtović‐Todorović et al., 1998), which is the opposite of the above‐described effect for oxygen. We hypothesize that both gases have different ligand‐binding sites on the NMDA receptor. Furthermore, nitrous oxide affects multiple other neuronal ion channel receptors (Mashour & Engelhard, 2019), and may differentially inhibit the same receptor at differing brain network locations. For example, “inhibiting the inhibitory” neurons will cause dis‐inhibition (i.e., excitation) effects, as has been shown with ketamine (Deane et al., 2020). This might explain why, despite both oxygen and nitrous oxide affecting the NMDA receptor oppositely, they both have a hyperexcitability effect on the whole brain.

On a more theoretical level, a reduction in DMN complexity is a decrease in recurrence entropy, because of a reduction in variation of recurrence that can be seen as a narrowing of the diagonal length histogram (figure 3c in Vrijdag et al., 2021). This phenomenon could indicate a more rhythmical brain state over the time scale of seconds to tens of seconds (Marwan et al., 2007). We can speculate that this decrease in temporal complexity is an early indicator of a shift in brain state toward an oscillatory instability that is a feature of a tonic–clonic seizure (Harrison et al., 2008). Seizures are one of the symptoms of central nervous system oxygen toxicity (Manning, 2016).

### 4.3 Strengths and limitations

A strength of this study was that we tested two hyperbaric oxygen exposure levels in a random order to counteract any learning effect on the psychometric test or a carry‐over effect. The first, at 142 kPa, resembles a regular oxygen exposure encountered in diving, while the exposure level at 284 kPa is the maximum oxygen exposure inside a dry hyperbaric chamber with an acceptable risk for oxygen toxicity. At both levels, we saw a similar degree of decrease in DMN complexity. The end‐tidal carbon dioxide measurement showed no sign of hypercapnia, which could have caused cognitive impairment. Hence, the influence of carbon dioxide retention can be excluded.

There were several limitations. First, the study involved a relatively small subject cohort, and was therefore susceptible to small sample bias. However, even in this small group, we showed a consistent decrease in the DMN complexity metric. Since we were looking for robust EEG changes, these changes needed to be visible in a small group to be of any value in the diving environment. The DMN complexity changes are similar in amplitude between 30% end‐tidal nitrous oxide and 100% oxygen. These results require further investigation and validation to understand their implications fully. This can be done by extending the dataset of oxygen exposures by replicating the study and by increasing the oxygen exposure time.

Second, the lack of significant change observed in the math processing task in the nitrogen narcosis study suggests a similar lack of sensitivity in the present study, unless oxygen proved to be more narcotic than nitrogen. This test is unsuitable for detecting subtle effects of gas narcosis. In future studies, we intend to use a more sensitive psychometric test.

Third, there was difficulty in interpreting the significant reductions in the subjective physical demand and effort scales of the NASA task load index during oxygen breathing periods at all pressures. It should be noted that the participants were not blinded to the gas and pressure they were breathing; hence these results could be caused by their expectations. It should also be noted that it would be hard to blind diver participants to pressure changes (Lansdorp & van Hulst, 2018).

## 5 CONCLUSION

Hyperbaric oxygen does not cause similar narcotic EEG effects to those induced by hyperbaric nitrogen. However, oxygen does seem to disturb the time evolution of EEG patterns in the default mode network when breathed at both normobaric and hyperbaric pressures. This could be a sign of oxygen‐induced neuronal hyperexcitability and requires further research.

## AUTHOR CONTRIBUTIONS

Xavier CE Vrijdag, Hanna van Waart, Simon J Mitchell, and Jamie W Sleigh designed the study. Hanna van Waart, Xavier CE Vrijdag, and Chris Sames collected data. Xavier CE Vrijdag, Hanna van Waart, and Jamie W Sleigh analyzed and interpreted the data. Xavier CE Vrijdag wrote the first draft. All authors reviewed and revised the manuscript. All authors have approved the final version of the manuscript and agree to be accountable for all aspects of the work. All persons designated as authors qualify for authorship, and all those who qualify for authorship are listed.

## FUNDING INFORMATION

This work has been supported by funding from the Office of Naval Research Global (ONRG), United States Navy (N62909‐18‐1‐2007).

## CONFLICT OF INTEREST

The authors declare no competing interests.

## ETHICAL APPROVAL

This study was performed in line with the principles of the Declaration of Helsinki. Approval was granted by the Health and Disability Ethics Committee, Auckland, New Zealand (8/9/2016, reference 16/NTA/93), and was registered with the Australian New Zealand Clinical Trial Registry (ANZCTR: U1111‐1181‐9722) on 12/03/2018. Written informed consent was obtained from all individual participants included in the study.

## Data Availability

The data that support the findings of this study are available from the corresponding author upon reasonable request. EEG analysis code is available online in previous publications.
